# Sequencing of five poultry strains elucidates phylogenetic relationships and divergence in virulence genes in *Morganella morganii*

**DOI:** 10.1186/s12864-020-07001-2

**Published:** 2020-08-24

**Authors:** Nicola Palmieri, Claudia Hess, Michael Hess, Merima Alispahic

**Affiliations:** Clinic for Poultry and Fish Medicine, Department for Farm Animals and Veterinary Public Health, University of Veterinary Medicine, Veterinärplatz 1, 1210 Vienna, Austria

**Keywords:** *Morganella morganii*, Poultry, NGS data, MALDI-TOF MS, Antimicrobial resistance, Virulence genes, Phylogeny

## Abstract

**Background:**

*M. morganii* is a bacterium frequently associated with urinary infections in humans. While many human strains are sequenced, only the genomes of few poultry strains are available. Here, we performed a detailed characterization of five highly resistant *Morganella morganii* strains isolated in association with *Escherichia coli* from diseased domestic Austrian poultry flocks, namely geese, turkeys and chicken layers. Additionally, we sequenced the genomes of these strains by NGS and analyzed phylogenetic clustering, resistance and virulence genes in the context of host-specificity.

**Results:**

Two strains were identified to be Extended Spectrum Beta Lactamase (ESBL) and one as AmpC beta-lactamases (AMP-C) phenotype, while two were ESBL negative. By integrating the genome sequences of these five poultry strains with all the available *M. morganii* genomes, we constructed a phylogenetic tree that clearly separates the *Morganella* genus into two clusters (M1 and M2), which approximately reflect the proposed subspecies classification (*morganii* and *sibonii*). Additionally, we found no association between phylogenetic structure and host, suggesting interspecies transmission. All five poultry strains contained genes for resistance to aminocoumarins, beta-lactams, colistin, elfamycins, fluoroquinolones, phenicol, rifampin and tetracycline. A comparative genomics analysis of virulence genes showed acquisition of novel virulence genes involved in secretion system and adherence in cluster M2. We showed that some of these genes were acquired by horizontal gene transfer from closely related *Morganellaceae* species and propose that novel virulence genes could be responsible for expansion of tissue tropism in *M. morganii*. Finally, we detected variability in copy number and high sequence divergence in toxin genes and provided evidence for positive selection in insecticidal toxins genes, likely reflecting host-related adaptations.

**Conclusions:**

In summary, this study describes i) the first isolation and characterization of *M. morganii* from goose and turkey, ii) a large-scale genetic analysis of *M. morganii* and an attempt to generate a global picture of the *M. morganii* intraspecific phylogenetic structure.

## Background

*Morganella morganii* is a facultative anaerobic Gram-negative enteric rod-shaped bacterium that used to belong to the *Enterobacteriaceae* family [1]. Recently, *Morganella* was suggested to be a type genus of a novel *Morganellaceae* family that consists of the following eight genera: *Arsenophonus*, *Cosenzaea*, *Moellerella*, *Morganella*, *Photorhabdus*, *Proteus*, *Providencia*, and *Xenorhabdus* [2]. *M. morganii* is reported as an opportunistic pathogen in humans and various other animal species [3]. In reported human cases the disease spectrum associated with *M. morganii* infections is broad and the mortality following an infection remains high [3]. In humans it has also been linked with animal bite wound infections, which underlines that *M. morganii* causes zoonotic infectious diseases [3]. However, the bacterium is scarcely reported in poultry: in Nigeria and China, *M. morganii* was isolated from dead broilers and its pathogenicity confirmed by infecting day-old chicks [4, 5]. Furthermore, *M. morganii* was isolated from chicken carcasses in US retail raw meat [6] and from a 13-day old broiler from a farm in Portugal [7]. Several factors can affect the progression and severity of an infection with antibiotic resistance as a main factor in both human and veterinary medicine. The World Health Organization has recently published a global priority list of antibiotic-resistant bacteria; “Priority 1: CRITICAL” ranking include extended spectrum β-lactamase (ESBL) producing and carbapenemase producing *M. morganii* (WHO 2019). *M. morganii* has a natural resistance to beta-lactam antibiotics (e.g. ampicillins, amoxicillin, most of the 1st and 2nd generation cephalosporins) and colistin [3]. In addition, the presence of pathogenicity determinants is essential to the success of *M. morganii* in any environment. Virulence genes cluster such as urease, flagellar and haemolysin were all found in *M. morganii* characterizing its potential as a pathogen and the ability to colonise different hosts [7–10]. In other *Enterobacteriaceae*, association between host and phylogenetic structure was observed in *E. coli* [11] but not in *Salmonella enteritidis* [12], neither in *Providencia* species [13], while the situation for *M. morganii* is presently unknown. A total of 47 complete genome sequences from *M. morganii* strains are currently available from NCBI database mainly isolated from humans, but also one from a broiler, salamander, dolphin, plants and few where the host is unknown. To date, a detailed genome analysis has been carried out for only few of these strains (KT, F675, INSRALV892a, MM 1, MM 4, and MM 190) [7–10] and detailed phylogeny is lacking. These studies reported peculiar strain-specific innovations such as: i) acquisition of resistance genes from *Acinetobacter* spp. in the F675 strain [9], ii) introduction of toxins (hlyA) through lateral transfer from phages in the MM1, MM4 and MM190 strains [10], and iii) plasmid-mediated acquisition of quinolone resistance in the INSRALV892a strain [7].

Here, we describe five poultry strains of *M. morganii* isolated for the first time from geese, a fattening turkey and a layer chicken parent flock. We characterized their resistance profile phenotypically and analyzed resistance genes, mobile elements and virulence genes through genome sequencing by Illumina NGS. By combining the genomic data of these poultry strains with all the available genomes we constructed a large-scale phylogeny of *M. morganii*, which we used to test the hypothesis of association between host and phylogeny and to highlight phenomena of lineage-specific divergence in virulence genes.

## Results

### Necropsy, bacteria isolation and identification

General fibrinous serositis was observed in dead birds from flocks A and B. In case of flock C, fibronecrotic typhlitis was the main gross pathological lesion found, a similar observation as in the 13-day old broiler from Portugal [7]. Omphalitis was diagnosed in turkey poults from flock D, while the chicken from a layer parent flock E did lack signs of bacterial infections but had a fracture of the leg. *Escherichia coli* was isolated from all cases investigated as reported by [7] who detected the same bacterium with *M. morganii* in liver and spleen of a broiler. *Riemerella anatipestifer* was detected in some organ samples from birds of flock B and *Clostridium perfringens* was found in association with a fibronecrotic typhlitis. When investigating the *E. coli* isolates for antibiotic resistance by disc diffusion method, smaller colonies were observed within the inhibition zone of ampicillin, amoxicillin and colistin, pointing towards a co-infection of *E. coli* and *M. morganii*. MALDI-TOF MS identified all strains to be *M. morganii* species, of which the strain PA17/10312 was classified as subspecies *sibonii* and the other four strains as subspecies *morganii* with a log score value above 2.5 (Table 1). On COS agar *M. morganii* could not be differentiated from *E. coli* or *Salmonella* spp., as both grow in round shape and appear as greyish smooth colonies. But on McConkey and Coliform agar, colonies were colourless and white (this could be due to the media), respectively, resembling those from *Salmonella* spp. Thus, morphological similarities to *E. coli* and *Salmonella* might have an impact on identification of *M. morganii* in routine diagnostics.

**Table 1:** Details on origin and identification results of *M. morganii* isolates

Flock	Strain Designation	Bird species	Age	Organ	Bacterial isolates	
A	PA17/10312	Goose	5 weeks	brain	*Morganella morganii* subsp. *sibonii, Escherichia coli*	
B	PA18/15564	Goose	10 weeks	heart	*Morganella morganii* subsp. *morganii, Escherichia coli, Riemerella anatipestifer*	
C	PA18/16407	Goose	40 weeks	heart, liver, intestine, lung	*Morganella morganii* subsp. *morganii, Escherichia coli, Clostridium perfringens*	
D	PA18/25921	Turkey	1 day	heart	*Morganella morganii* subsp. *morganii, Escherichia coli*	
E	PA19/9695	Layer parent stock	32 weeks	heart	*Morganella morganii* subsp. *morganii, Escherichia coli*	

### Antibiotic susceptibility testing

All isolates were found to be multidrug resistant (resistance to more than three antimicrobial substances) by disc diffusion method with resistance patterns ranging from five to ten antibiotics (Table 2). All were resistant to amoxicillin, ampicillin, and colistin, but also to tilmicosin and tylosin. The combination of trimethoprim/sulfamethoxazole was the only antibiotic to which all five strains were susceptible. Resistance to β-lactam antibiotics in *Morganella* species is usually mediated by the presence of chromosomally encoded β-lactamases belonging to the AmpC β-lactamase family [14]. These β-lactamases are typically induced in the presence of β-lactam antibiotics. The strain PA17/10312 (flock A) was confirmed to be an AMP-C phenotype. In contrast to ESBLs, AmpC hydrolyses broad and extended-spectrum cephalosporins (cephamycins as well as to oxyimino-β-lactams) but are not inhibited by β-lactamase inhibitors such as clavulanic acid [15]. The strains PA18/15564 (flock B) and PA18/25921 (flock D) were confirmed to be ESBL phenotype and possible productors of type D carbapenemases or impermeability/porin loss, whereas the strains PA18/16407 (flock C) and strain PA19/9695 (flock E) were ESBL negative.

**Table 2:** Antibiotic susceptibility testing by disc diffusion method – R = Resistant, S = Susceptible, I = Intermediate

Antibiotics (μg)	PA17/10312	PA18/15564	PA18/16407	PA18/25921	PA19/9695
Amoxicillin (10)	R	R	R	R	R
Ampicillin (10)	R	R	R	R	R
Colistin (10)	R	R	R	R	R
Doxycycline (30)	R	I	R	R	S
Enrofloxacin (5)	S	I	R	R	R
Neomycin (30)	I	I	I	S	S
Oxalic Acid (2)	S	S	S	R	R
Spectinomycin (100)	S	S	S	R	S
Tetracycline (30)	I	I	R	R	S
Tilmicosin (15)	R	R	R	R	R
Trimethoprim/Sulfamethoxazole (25)	S	S	S	S	S
Tylosin (150)	R	R	R	R	R

### Sequencing and annotation of five poultry strains

**Fig. 1:**
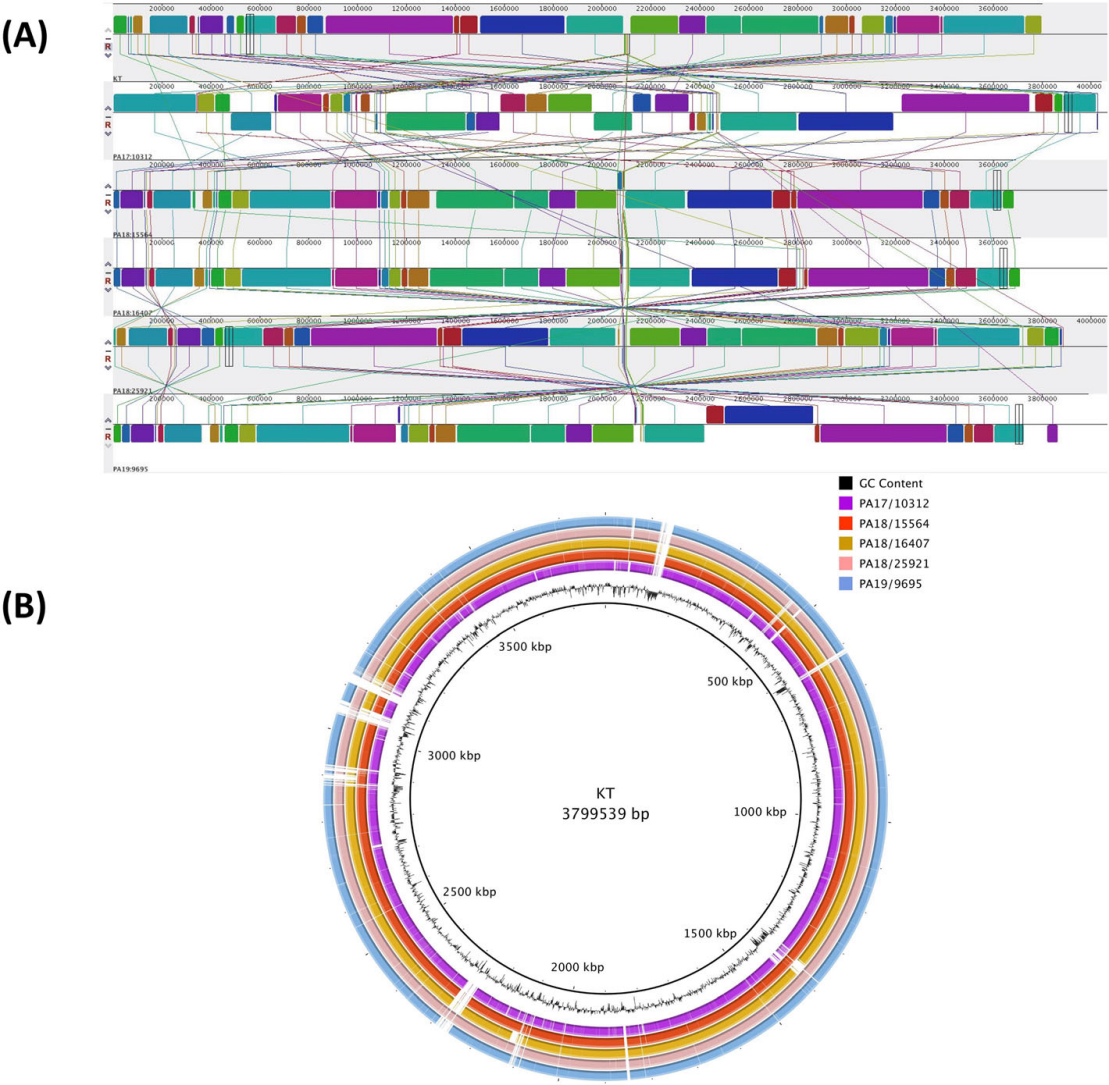
**a** Multiple genome alignment of the five sequenced poultry strains and the KT reference strain **b** Circular genomic map constructed using BRIG displaying a multiple genome alignment of the five poultry strains and the KT reference strain

The genome sequences of the five poultry strains displayed substantial variation in length and number of genes (Table 3), with the PA17/10312 being longer and having more genes compared to the other strains. To evaluate whether these differences might be due to missing annotations, we computed genome completeness using the program CheckM [17] for each assembly and obtained values of 100% for all five strains, implying that the observed variation in gene content cannot be attributed to uneven coverage of the assembled strains. Genome size and gene content were also comparable to the assemblies of other published strains [7–10]. Additionally, we observed variations in genome structure among the five strains (Fig. 1a): PA18/15564 and PA18/16407 showed high colinearity compared to the closest reference strain (Additional file 2: Supplementary Figure 1A), while PA17/10312 displayed more genomic rearrangements (Additional file 2: Supplementary Figure 1B). Finally, the genome of the five strains was aligned to the reference strain KT, for which the most comprehensive annotation is available [8]. A multiple alignment in circular form is displayed in Fig. 1b and shows a high level of conservation among the five poultry strains, with few shared gaps, corresponding to the rRNA ribosomal gene clusters, which are notoriously difficult to assemble due to their repetitive nature.

**Table 3:** Genomic features of the five sequenced poultry strains

	PA17/10312	PA18/15564	PA18/16407	PA18/25921	PA19/9695
Number of contigs	78	59	47	548	667
Total length (bp)	4,085,866	3,675,879	3,702,167	4,066,432	3,988,823
N50 (bp)	244,109	403,330	989,625	403,126	49,931
GC content	50.2%	51.2%	51.2%	51.0%	51.6%
Genes	3840	3452	3490	3812	3756
Coding genes	3784	3393	3431	3718	3671
Genome completeness	100%	100%	100%	100%	100%
Contamination score (%)^a^	0.00	0.54	0.00	1.92	2.75
Strain heterogeneity^b^	0.00	0.00	0.00	37.50	50.00

^a,b^ For a detailed description of these metrics, see the CheckM paper [16]

^b^ The strain heterogeneity (SH) index indicates the proportion of the contamination that appears to be from the same or similar strains and is a number between 0 and 100

### Phylogenetic analysis

**Fig. 2:**
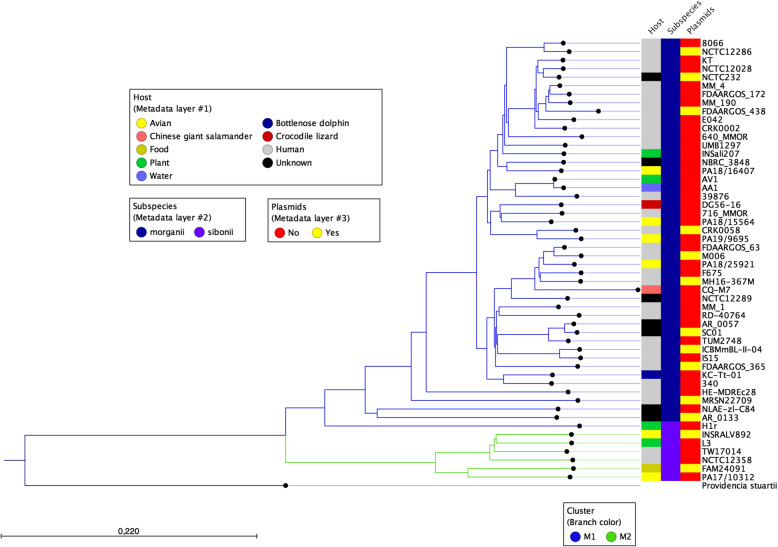
K-mer tree including the five sequenced poultry strains and 47 complete genomes from NCBI, using *Providencia stuartii* as an outgroup

To test the hypothesis of a phylogenetic clustering based on the host in *M. morganii*, a phylogenetic tree was constructed including the five sequenced poultry isolates together with all available complete genomes from NCBI, encompassing a total of 52 strains. The genome-based k-mer tree displayed two distinct subtrees (M1 and M2), with no apparent clustering according to the host they were isolated from (Fig. 2). The resulting tree places the strains PA18/16407, PA18/15564, PA19/9695, PA18/25921 in cluster M1 and the PA17/10312 strain in cluster M2. Historically, *M. morganii* has been divided into two subspecies: *morganii* and *sibonii*, based on the ability to ferment trehalose and other biochemical features [18]. Thus, we hypothesized that clusters M1 and M2 simply reflect the known subspecies classification. To test this idea, the presence of the trehalose operon (*treR*, *treB* and *treP*) in all the strains was investigated. By this approach, the subspecies *sibonii* was assigned to the strains in which the trehalose operon was present. Comparing this classification to the observed phylogenetic clustering, we found that the sibonii strains were entirely allocated in cluster M2, except the H1R strain (Fig. 2), confirming that differentiation of subspecies can be mostly performed based on phylogenetic grouping. These results are consistent with the subspecies assignment achieved by MALDI-TOF MS (Table 1). To validate the accuracy of the k-mer tree reconstruction, an additional tree was built using a whole-genome based approach using the program parsnp (Additional file 3: Supplementary Figure 2A). Comparison of both trees showed high similarity in topology with only minor differences: i) the NCTC12358 and TW17014 strains of cluster M2 are swapped in the whole-genome based tree compared to the k-mer tree; ii) the 640_MMOR strain is grouped together with NCTC12286 and 8066 in the whole-genome based tree, while in the k-mer tree constitutes a separate cluster. Since recombination can sometimes distort branch lengths in bacterial whole-genome based phylogenies (i.e. [19]), we performed another tree reconstruction by filtering SNPs located in putative regions of recombination (parsnp parameter -x YES) (Additional file 3: Supplementary Figure 2B). However, we did not find significant differences in branch length compared to the uncorrected tree (Mann-Whitney test – *P* = 0.9914). As the differences between the whole-genome based trees and the k-mer tree do not affect the conclusions derived from following analyses, the k-mer tree was used as a guide for the remainder of the analyses.

### Detection of resistance genes

**Fig. 3:**
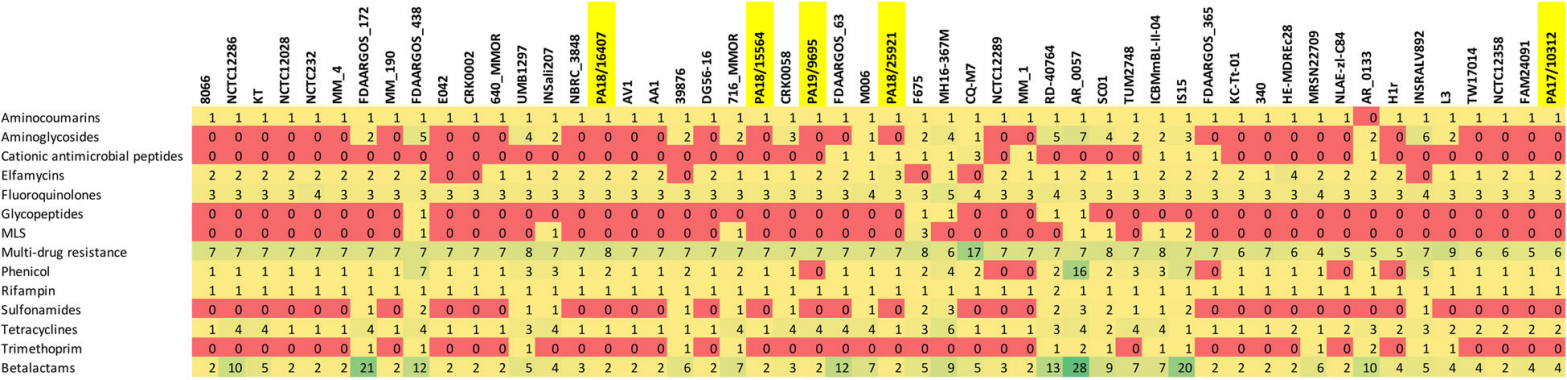
Phylogenetic distribution of resistance genes in the 52 analyzed *M. morganii* strains ordered according to the position in the tree from Fig. 2. The five sequenced poultry strains are highlighted in yellow. Every cell contains the number of gene copies involved in a certain type of resistance for each strain. Cells are coloured using conditional formatting to facilitate readability

The sequences of all five poultry strains contained genes for resistance to aminocoumarins, beta-lactams, elfamycins, fluoroquinolones, rifampin and tetracycline. Resistance to phenicol was present in all the sequenced poultry strains, except in the chicken strain PA19/9695. Based on BLAST searches against the MEGARes database, the investigation of presence/absence of resistance genes in all 52 strains from Fig. 2 revealed that resistance to certain antibiotics is widespread in *Morganella*. This includes aminocoumarins, beta-lactams, elfamycins, fluoroquinolones, phenicol, rifampin and tetracyclines (Fig. 3). Other types of resistance show a more uneven distribution along the tree, with multiple cases of strain-specific gains, like resistance to glycopeptides, which is present in 5/52 strains with no apparent phylogenetic clustering. This suggests that these genes could have originated by horizontal gene transfer. Additionally, we looked at resistance genes that were absent in the MEGARes database, but that were previously reported for other strains of *M. morganii*, such as genes involved in lipid A biosynthesis (*lpxA*, *lpxB*, *lpxC*, *lpxD*, *lpxH*, *lpxK*, *lpxL*, *lpxM, lpxP* and *lpxT*), which are responsible for colistin resistance [9]. Such analyses detected *lpxABCDHKLMPT* in both geese strains, *lpxABCDHKLMT* in the chicken strain PA19/9695, *lpxABCDHKLMT* in the turkey strain PA18/25921 and *lpxABCDHLMT* in the goose strain PA17/10312. An additional copy of the *lpxP* gene was found in PA18/25921 and PA17/10312. Also, the *tetB* gene responsible for tetracycline resistance was found in all five poultry strains. No association was detected between resistance genes of a certain class and a specific phylogenetic cluster. These results are concordant with the phenotypic tests of antibiotic resistance (Table 2).

### Detection of known virulence genes

**Fig. 4:**
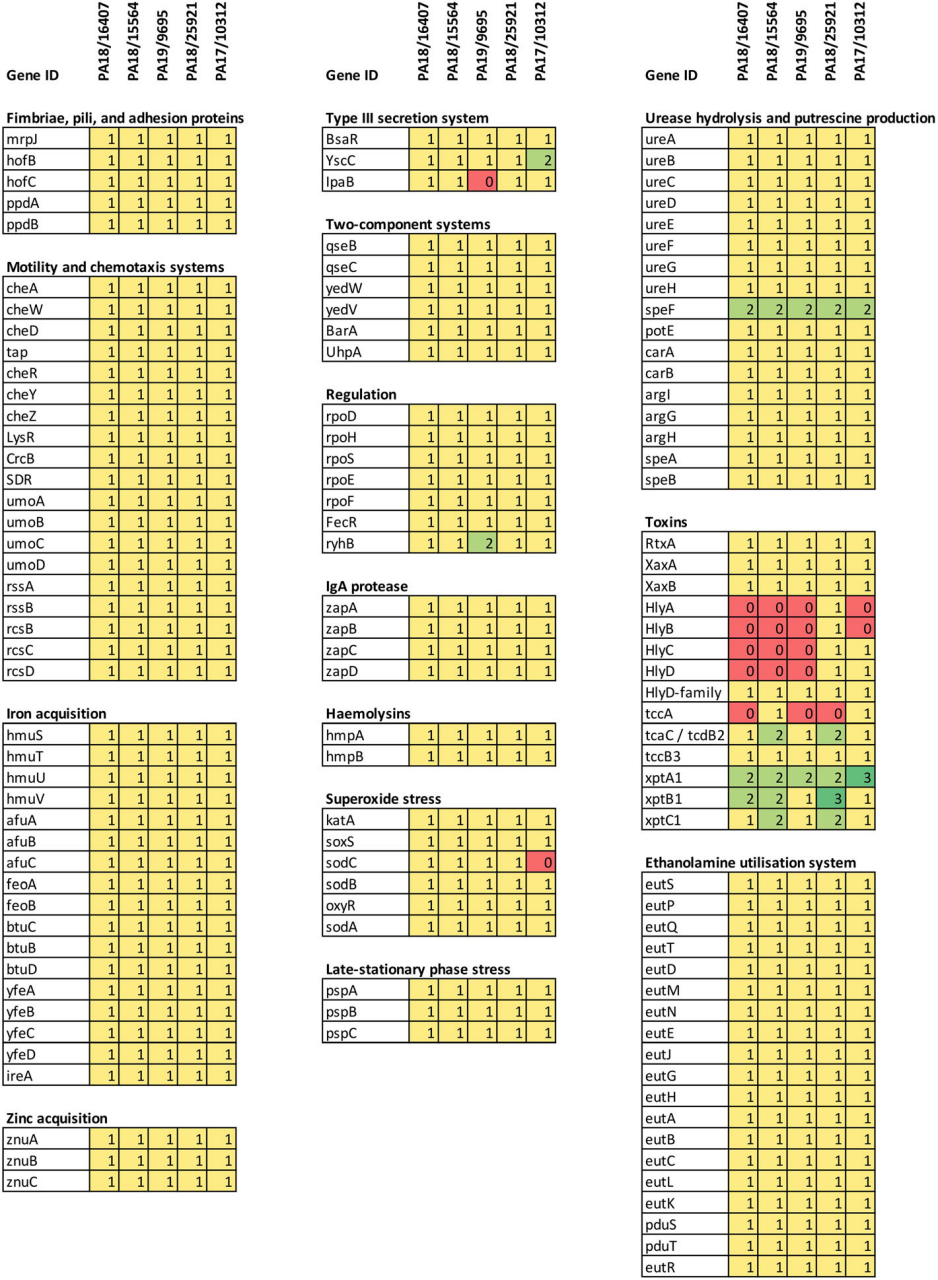
Comparison of known virulence genes in the five sequenced poultry strains using the KT strain as reference

In an initial approach, the sequences from the poultry strains were screened for known virulence genes based on the comparison with the KT strain, for which an exhaustive list of virulence genes is available. However, the gene-id based detection of genes from the KT strain was unfeasible due to incongruences between gene-ids from the KT strain and the ones deposited in the NCBI gene database, thus the 4-letter gene name was used to retrieve the genes of interest, resulting in a partial set of 125 genes for this analysis. Most of the known virulence genes present in this set were found in the sequences of the five poultry strains (Fig. 4). This not only indicates that virulence genes are very conserved, but also suggests that the annotations of the five poultry strains is highly complete. As this gene set did not include all known virulence genes, we additionally looked for the presence of specific classes of virulence genes of importance. Hemolysins are essential for pore-formation during invasion of host cells [20] and encoded by the *hlyCABD* operon. This operon was present and intact only in the chicken strain PA18/25921, while absent in PA18/16407, PA18/15564 and PA19/9695. In the sibonii strain PA17/10312, only *hlyC* and *hlyD* were present and distributed on different contigs. Ureases are also typically organized into the *ureABCFGD* operon and contribute to formation of urinary stones [21]. This operon was present and intact in all five strains. Finally, the capsule synthesis regulation genes *rcsB*, *rcsC*, *rcsD* and *rcsF* involved in host immune response [22] were also detected in all five strains, with an additional copy of *rcsB* in PA18/25921. Interestingly, virulence genes in the category “toxins” displayed high variation in copy numbers among the five strains (Fig. 4). Manual scanning of the alignments also showed substantial variation in coding sequences exemplarily shown for the RtxA toxin in Additional file 4: Supplementary Figure 3. For these reasons, a positive selection scan using PAML (see Materials and methods) was performed based on the hypothesis that toxins might evolve under positive selection in *M. morganii*. For this analysis, only toxins present in all five strains were selected (Fig. 4) and it was found that four out of nine toxins (*tcaC*/*tcdB2*, *xptA1*, *xptB1*, *xptC1*) showed signs of positive selection using branch models by selecting either cluster M1 or M2 as the target branch (Table 4). Notably, the insecticidal proteins *XptA1B1C1* had the strongest signal of positive selection.

**Table 4:** List of toxins and associated *p*-values for the positive-selection test using cluster M2 or M1 as target branch. Significant genes are highlighted in grey

Toxin	***P***-value M2	***P***-value M1
RtxA	1.0000	1.0000
XaxA	0.9980	0.6633
XaxB	0.9546	1.0000
HlyD-family	1.0000	1.0000
tcaC / tcdB2	0.0200	0.0200
tccB3	1.0000	1.0000
XptA1	0.0003	0.0006
XptB1	0.0010	0.0010
XptC1	0.0036	0.0009

### Detection of novel virulence genes

**Fig. 5:**
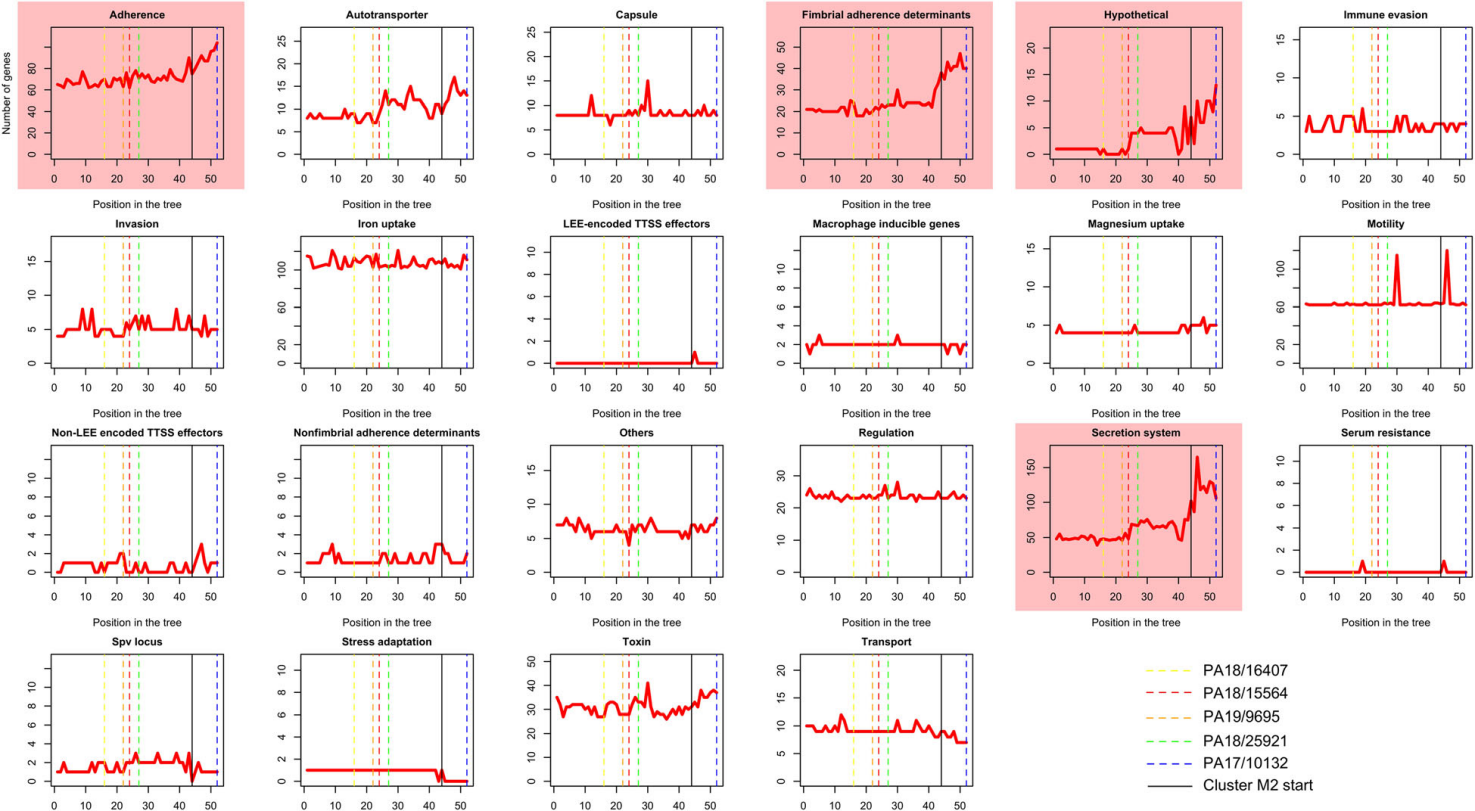
Phylogenetic distribution of virulence genes of different virulence categories as defined from the VFDB – each graph shows the number of genes per strain for every virulence category. Axes labels are shown in the top-left graph only – strains are sorted according to the phylogenetic position on the tree in Fig. 2. The position of the five avian strains in each graph is highlighted by vertical dashed lines of different colours. Graphs showing variation between clusters M1 and M2 are highlighted by a pink background

To get an overview of the variation in individual virulence genes across the strains, the number of virulence genes of different functional categories in each strain was plotted and sorted according to the phylogenetic position on the tree (Fig. 5). An increase in number of genes in strains of cluster M2 compared to the cluster M1 in the virulence categories adhesion, secretion system and hypothetical was observed (Fig. 5). As the strain PA17/10312 is also located in cluster M2, these results might explain the higher number of genes in the PA17/10312 strain compared to the other strains. Novel virulence genes were defined as those specific to cluster M2: a total of 39 novel virulence genes were found in this way (Table 5). To validate specificity to cluster M2, these genes were BLASTed to two outgroup species: *Providencia stuartti* and *Proteus mirabilis*, and only genes with no hits to both species were retained. This resulted in a final set of 26 novel virulence genes. Regarding phylogenetic distribution, 10/26 genes were shared by all strains in cluster M2, consistent with ancestral gain in cluster M2 and 12/26 genes were specific to a single strain, likely due to strain-specific horizontal gene transfer events. To find evidence for horizontal gene transfer the novel virulence genes were BLASTed against the whole bacterial protein database. Then, using the program Alienness [23], evidence for horizontal gene transfer (HGT) was detected in 6/26 (23%) of the novel virulence genes (*afaB, escU, escV, SG1030, stx2A, ycbV*), having an Alien Index (AI) > 30 (Additional file 5: Supplementary Table 2). These genes were assigned to the different donors of the class *Morganellaceae* (Additional file 5: Supplementary Table 2).

**Table 5:** List of novel virulence genes specific to the M2 cluster

Category	Genes
Adherence	*afaAB, eae, fdeC, pagN, papADF, pixACH, sfaG, sfpAC, stiC, stkB, ycbV*
Secretion system	*aatA,* EC55989_3333*,* ECP_0235*,* ECVR50_0257*, escNSTUV, espD,* LF82_024*, mxiH,* O3M_04340*, pipB, prgI, sipB*
Hypothetical	SG1030
Toxins	*clbN, stx2A, stxA*
Iron uptake	*fyuA*
Others	STM0570

### Prediction of pathogenic potential for humans

Using PathogenFinder 1.1 [24] the pathogenic potential of the five poultry strains for humans was estimated. The approach used by this tool is based on the presence of group of genes that are frequently associated with human pathogenic bacteria. Results showed that the PA17/10312 was predicted to be pathogenic in humans (Probability = 0.64), while the four other strains were classified as non-pathogenic in humans (Probability = 0.57 for PA18/15564, 0.52 for PA18/16407, 0.57 for PA18/25921 and 0.54 for PA19/9695). These results are consistent with the higher number of virulence factors found in PA17/10312 compared to the other strains. It remains to be determined in an animal trial whether these findings also reflect the host-specific pathogenicity.

### Annotation of mobile elements

Mobile elements such as plasmids, prophages and integrons are often responsible for transfer of resistance genes in bacteria [25, 26]. No evidence for plasmids was found in any of the five poultry strains using two independent approaches (see Materials and methods). As plasmid information is only available for some of the published strains (i.e. [7, 8, 27–29]), we screened all the strains of Fig. 2 using PlasmidFinder [16] for the presence of plasmids and results are shown in Fig. 2 (Metadata layer 3). A total of four active prophages were detected in PA17/10312, three in PA18/15564, two in PA18/16407, five in PA18/25921 and one in PA19/9695 (Additional file 6: Supplementary Table 3), none of them showing association with resistance genes. Two integrons were found in the PA19/9695 strain and one integron was found in the PA17/10312 strain, also not associated with resistance genes. Pathogenicity islands are another class of mobile elements, which are linked with virulence genes in a large number of bacteria [30]. A total of 18 pathogenicity islands were detected in PA17/10312, 16 in PA18/15564, 21 in PA18/16407, 22 in PA18/25921 and 23 in PA19/9695 (Additional file 7: Supplementary Table 4, Additional file 8: Supplementary Figure 4), associated, respectively, with 25, 9, 23, 16 and 24 virulence genes. The most abundant virulence categories associated with these genes were: “adherence” for 20/25 genes in PA17/10312, “adherence” for 5/9 genes in PA18/15564 and “iron uptake” for 11/23 genes in PA18/16407, “toxins” for 5/16 genes in PA18/25921 and “adherence” for 11/24 in PA19/9695. A striking feature of pathogenicity islands is that they are located in regions of low genomic conservation, (Additional file 8 – Supplementary Figure 4), suggesting that they were acquired by phenomena of lateral gene transfer.

### Identification of strain-specific metabolic pathways

To study metabolic pathways, the proteomes of the five poultry strains were submitted to BlastKOALA [31], which provides a global overview of metabolic pathways displayed as a metabolic network. This program also allows an easy comparison of metabolic networks among different organisms or strains. All metabolic pathways were entirely conserved among the five poultry strains. In addition, two novel pathways specific to the PA17/10312 strain were discovered (Additional file 9: Supplementary Figure 5): sucrose catabolism and biosynthesis of betaine from choline. Betaine is an osmoprotectant that allows organisms to grow in high-osmolarity environments [32]. Some bacteria can synthesize betaine de novo from intermediates of central metabolism whereas others can synthesize it only from exogenously supplied choline [33]. Screening all the 52 *M. morganii* strains included in Fig. 2 revealed that only the FAM24091 strain (isolated from Switzerland cheese), which is the closest strain to PA17/10312, possess genes for betaine biosynthesis (BetA and BetB). No evidence for horizontal gene transfer was found, based on the absence of proteins from closely related species which would cluster together with BetA and BetB from PA17/10312 based upon BLAST analyses. Whether the determined pathways are beneficial for the bacteria in vitro and in vivo remains speculative for the moment and needs to be determined in additional studies.

## Discussion

In the last years, many studies isolated and sequenced *Morganella morganii* strains from human [8–10, 27–29] while the genomic characterization for other species is scarce and include a strain from chicken [7], cattle [34], together with strains from various hosts that were deposited in NCBI but not described in details (see Fig. 2). Here, we expanded the collection of avian *M. morganii* by sequencing five novel strains from goose, turkey and chicken. Understanding the relationship between host and phylogeny is useful to study population structure and uncover the origin and evolution of novel strains, in order to understand the emergence of potential outbreaks. Overall, the phylogenetic analysis showed no association between host and a particular phylogenetic cluster, consistent with previous studies also reporting lack of association with host in *M. morganii* [27, 34]. This characterizes *M. morganii* as a species with no specific population structure. Regarding the subspecies classification, we found that four of the avian strains (PA18/15564, PA18/16407, PA18/25921 and PA19/9695) were assigned as subsp. *morganii*, while the strain PA17/10312 was assigned to subsp. *sibonii* based on phylogenetic clustering. When looking at the tree including all the available strains, we found that one strain (H1r) classified as *sibonii* based on the presence of the trehalose genes was assigned to cluster M1, implying that the characterization of subspecies in *M. morganii* purely based on the presence of the trehalose fermentation genes or the phylogenetic clustering pattern alone can sometimes be imprecise. Antibiotic resistance is another important feature of clinical relevance, in this regard it was previously shown that another avian *M. morganii* strain was multidrug resistant [7], raising concerns for the control of infections in poultry production. In this study, we found that all five strains were resistant to aminocoumarins, beta-lactams, elfamycins, fluoroquinolones, rifampin and tetracycline. Importantly, these classes of resistance were also widespread in all sequenced strains from Fig. 2. The antibiotic resistance results are in agreement with earlier data describing ESBL producers as multiple drug resistant [35], also the classes of resistance determined computationally were consistent with a previously compiled review of resistance genes [3]. Additionally, the genomic characterization allowed us to compare presence of specific virulence genes associated with certain strains or phylogenetic clusters. Within the essential virulence genes, ureases and capsule synthesis regulation genes were very conserved in all five strains, while haemolysins showed more variability. Variation in hemolysin composition was also observed in three recently sequenced human strains [10]. A general pattern emerged by looking at the distribution of virulence genes among all sequenced strains, namely we observed that strains of the subspecies sibonii had on average more virulence genes compared to the subspecies morganii and were particularly enriched in virulence genes involved in secretion and adhesion. These results were also corroborated by the prediction that *sibonii* strains were found to be potentially pathogenic for human. Through comparative genomics analysis, we pinpointed 26 virulence genes as specific to the subspecies *sibonii* and showed that some of them might have originated by horizontal gene transfer from closely related Morganellaceae species. Among these genes, *afaAB* [36], *pixC* [37], *sfaG* and *fyuA* [38] are known to be associated with adhesion and colonization of urinary tract in *E. coli*, while the intimin *fdeC* [39], the Shiga toxins *stx2A*/*stxA* [40], *escN* [41], *espD* [42] are connected with enteropathogenic strains of *E. coli*. This suggests that some *M. morganii sibonii* strains might have acquired the ability to invade the enteric tract. Another important classes of virulence genes are toxins, for which we showed evidence for positive selection, especially high in the insecticidal toxins *XptA1B1C1.* While the precise function of these toxins in *M. morganii* is unknown, it was shown in *Xenorhabdus spp*. and *Photorhabdus luminescens* that they facilitate the killing of the insect host via nematodes living in symbiosis with these bacteria [43]. Thus, positive selection in insecticidal toxins might reflect an evolutionary arms race among species.

## Conclusion

In the actual study, five highly resistant *Morganella morganii* strains from diseased poultry flocks were isolated in association with *E. coli*. The strains were identified by MALDI-TOF MS down to subspecies level. Micronaut characterization revealed two strains to be ESBL and one strain to be AMP-C phenotypes. To gather more detailed information about their association with the host, resistance profile, virulence and zoonotic potential, the genomes of the five strains were sequenced and compared with all available complete *M. morganii* genomes. The phylogenetic structure of *M. morganii* separates all strains into two main phylogenetic clusters (M1 and M2) that approximately reflect the subdivision of the *Morganella* clade into the known subspecies *morganii* and *sibonii*, with no association between phylogenetic structure and host. Additionally, we showed that cluster M2 diverged through acquisition of novel virulence genes involved in adherence and secretion and that some of these genes might have originated by horizontal gene transfer from closely related *Morganellaceae* species. Furthermore, high sequence divergence in toxins was detected and evidence for positive selection for some of these genes was provided. Finally, we reported detailed resistance profiles for all so far sequenced *M. morganii* strains complementing the phenotypic resistance profile of the five poultry strains. Altogether, this study represents the first large-scale comparative study in *M. morganii* and represents a useful resource to aid future clinical management of infections and for advancing the field of comparative genomics of *Morganella*. Performing experimental studies will be crucial to further clarify the nature of *M. morganii* as an avian pathogen and its zoonotic potential.

## Methods

### Necropsy, bacteria isolation and identification

During routine diagnostics the Clinic of Poultry and Fish Medicine (Vetmeduni Vienna, Austria) obtained carcasses from three geese flocks (A-C), one-day old turkey (D) and one-layer chicken parent (E) flocks (Table 1) for necropsy and bacteriological investigation. Necropsy and bacteriology were performed in the diagnostic unit of the clinic accredited according to EN ISO/IEC 17025 applying standard operating procedures. For bacteriological investigation, direct smears were taken aseptically from heart, liver, intestine, brain, spleen and lungs. Samples from each organ were cultivated on Columbia (COS) agar containing 5% sheep blood (BioMerieux, Vienna, Austria), McConkey agar (Bertoni, Vienna, Austria), Chromocult Coliform agar (Merck, Vienna, Austria) and subsequently incubated aerobically at 37 °C for 24 h. Additionally, intestinal samples were cultivated on Schaedler (SCS) agar containing 5% sheep blood (BioMerieux, Vienna, Austria) for anaerobic growth (Genbox anaer BioMerieux) incubated at 37 °C for 24 h as well as for fungal growth on Sabouraud Gentamicin Chloramphenicol (SGC2) agar (BioMerieux, Vienna, Austria) incubated at 42 °C for 48 h. All bacterial isolates were identified by Matrix Assisted Laser Desorption/Ionization-Time of Flight Mass Spectrometry (MALDI-TOF MS) (Microflex LT instrument, Bruker Daltonic) against the reference library from Bruker Daltonic version 4.1.80 according to the manufacturer’s instructions, with sample preparation performed as previously described [44].

### Antibiotic susceptibility testing

Antibiotic susceptibility testing was performed by disc diffusion method according to the EUCAST guidelines ([45], EUCAST disc diffusion method, version 6.0, 2019. http://www.eucast.org/clinical_breakpoints/). Phenotypic detection of ESBL type A β-lactamases, AmpC type C cephalosporinases, KPC type A carbapenamases and MBL metallo-β-lactamases was performed by broth microdilution method using the commercially available MICRONAUT-S ß-Lactamases plateE1–111-040 (MERLIN Diagnostika GmbH, Bornheim-Hersel, Germany). The antimicrobial substances and their concentration are given in Additional file 1: Supplementary Table 1. The analysis was done according the manufacturer’s instructions. Results were evaluated with the MCN6 Software Version 6.00 Release 71 (MERLIN Diagnostika GmbH, Bornheim-Hersel, Germany).

### Genome sequencing and annotation

DNA was extracted using QIAamp DNA Mini Kit following the manufacturer’s guidelines (Qiagen, Hilden, Germany). The five samples were sent to the Vienna BioCenter Core Facility, where they were sequenced on an Illumina NextSeq machine using a paired-end PCR-free library (150 bp read length). Resulting reads were assembled into contigs using CLC Genomics Workbench 12.0 (https://www.qiagenbioinformatics.com/) by the De Novo Assembly workflow (default parameters) and annotated using PROKKA 1.13 [46]. Plasmids were detected using PlasmidFinder 2.0 [16] and by BLAST searches against a local version of the *Enterobacteriaceae* plasmid database [47]. Prophages were annotated using the recent tool Prophage Hunter [48], which is also able to differentiate active from inactive prophages. Only active prophages were selected into the final annotation. Integrons were annotated by IntegronFinder 1.5.1 [49]. Pathogenicity islands were identified by IslandViewer 4 [50] using the *M. morganii* KT strain [8] as a reference.

### Phylogenetic trees construction

For tree construction, the five sequenced assemblies were employed together with all 47 complete *M. morganii* genomes downloaded from NCBI (see Additional file 10 – Supplementary Table 5 for a list of GenBank accession IDs) encompassing a total of 52 strains. For each strain, the genome sequence was concatenated to form a single artificial sequence, including plasmids and unscaffolded sequences. A k-mer phylogenetic tree was constructed using CLC Microbial Genomics 12.0 by the Create K-mer Tree workflow (default parameters). Additional phylogenetic trees were constructed based on multiple genome alignment of all the strains with the program parsnp [51], using the KT strain [8] as a reference.

### Identification of resistance genes

The protein sequences of each strain were BLASTed (blastp -evalue 1e-05) to a local version of the MEGARes database [52], which contains more than 4000 hand-curated antimicrobial resistance genes. Hits with a query coverage > 40% were kept for further analyses. Additional screening for resistance genes that were absent in the MEGARes database (*lpx* genes, *tetB*) was performed using the same BLAST-based approach.

### Identification of virulence genes

The protein sequences of virulence genes for the *M. morganii* KT strain [8] (GenBank ID GCA_000286435.2) were retrieved using the 4-letter gene-id from the NCBI Protein database and BLASTed (blastp -evalue 1e-05) to the proteomes of the five sequenced poultry strains. Hits with a query coverage > 40% were kept and processed. Additional virulence genes were identified by BLASTing the protein sequences of the five strains to a local version of the VFDB (Virulence Factor DataBase) using stringent criteria (blastp -evalue 1e-10, query coverage > 40%), in order to get a conservative set of virulence genes. Novel genes were detected in the following way: the proteomes of the 52 strains of *M. morganii* from Fig. 2, together with the representative NCBI strain of *P. stuartii* and *P. mirabilis* (as outgroups) were BLASTed to the VFDB with stringent criteria (blastp -evalue 1e-10, query coverage > 40%) and a table was generated containing the phyletic patterns of each virulence gene in every strain. Novel genes were then defined as genes-specific to cluster M2 (see Fig. 2). Annotation of functional classes for virulence genes was based on the intra-genera VFs comparison tables from VFDB (http://www.mgc.ac.cn/VFs/Down/Comparative_tables_from_VFDB.tar.gz). Gene families were computed by clustering the protein sequences of each strain using the sequence clustering program MMseqs2 [53] with a minimum 80% similarity threshold.

### Positive-selection scans

Coding sequences for orthologous groups of genes of interest were aligned using PRANK [54]. Positive selection scans were performed with the codeml module from PAML [55] by comparing a model with a fixed ω for the whole tree (model = 2, NSsites = 2, fix_omega = 1) with a model allowing positive selection in the M2 cluster (Fig. 2) (model = 2, NSsites =2, fix_omega = 0) or in the M1 cluster (model = 2, NSsites =2, fix_omega = 0). *P*-values were calculated by standard log-likelihood ratio tests.

### Identification of strain-specific metabolic pathways

Protein sequences were submitted to BlastKOALA [31] to reconstruct the metabolic network of each strain. The metabolic networks of the different strains were compared through the KEGG Pathway interface from the KEGG database [56] in order to discover strain-specific pathways.

## Supplementary information

**Additional file 1: Table S1.** MICRONAUT antimicrobial substances and concentrations.

**Additional file 2: Figure S1.** Genomic alignment computed with MAUVE between two of the sequenced poultry strains and the closest strain on the tree shown in Fig. 2: **(A)** PA18/15564 aligned to the DG56–16 strain, **(B)** PA17/10312 aligned to the INSRALV892 strain.

**Additional file 3: Figure S2. A)** Tree of the 52 *M. morganii* strains analyzed in this study derived by alignment of the core genome using the program *parsnp*. The KT strain was used as a reference for the multiple genome alignment. **B)** The same tree but corrected for recombination. The red arrows indicate the cluster that shows differences between the two trees.

**Additional file 4: Figure S3.** Multiple alignment of an internal region of the RtxA toxin in the five poultry strains showing high sequence divergence.

**Additional file 5: Table S2.** List of candidate genes in the PA17/10312 strain with evidence for horizontal gene transfer from closely-related *Morganellaceae* species.

**Additional file 6: Table S3.** Annotation of active prophages in the five sequenced poultry strains.

**Additional file 7: Table S4.** Annotation of pathogenicity islands in the five sequenced poultry strains. Predicted genomic islands are coloured within the images based on the legend (generated through the web interface of IslandViewer 4 [50]). The internal line plot shows the similarity to the KT strain, taken as a reference.

**Additional file 8: Figure S4.** Genomic visualization of pathogenicity islands in the five sequenced poultry strains, performed in this study.

**Additional file 9: Figure S5.** Metabolic network of *M. morganii* strain PA17/10312 (computed through the web interface of BlastKOALA [31] in this study) showing two novel pathways specific to this strain: **(A)** Sucrose catabolism, sucrose = > glucose, **(B)** Betaine biosynthesis, betaine = > choline.

**Additional file 10: Table S5.** List of *M. morganii* strains used for the phylogenetic analysis with their respective NCBI accession IDs.

## Data Availability

The datasets generated and/or analyzed during the current study are available in the NCBI repository (Accession IDs of the five sequenced poultry strains: WCIS00000000, WCIT00000000, WCIU00000000, WCIV00000000, WCIW00000000). The list of *M. morganii* strains used for the phylogenetic analysis and their respective NCBI accession IDs are listed in Supplementary Table 5.

## Abbreviations

AI: Alien Index
AmpC: AmpC beta-lactamases
BLAST: The Basic Local Alignment Search Tool
COS: Columbia agar
ESBL: Extended-spectrum beta-lactamases
EUCAST: The European Committee on Antimicrobial Susceptibility Testing
HGT: Horizontal Gene Transfer
MALDI-TOF MS: Matrix Assisted Laser Desorption/Ionization-Time of Flight Mass Spectrometry
NGS: Next Generation Sequencing
SCS: Schaedler agar
SGC2: Sabouraud Gentamicin Chloramphenicol agar
SNPs: Single nucleotide polymorphisms

## References

[1] O’Hara CM, Brenner FW, Miller JM. Classification, identification, and clinical significance of Proteus, Providencia, and Morganella. Clin Microbiol Rev. 2000;13:534–46. 10.1128/cmr.13.4.534-546.2000 PMC88947 11023955

[2] Adeolu M, Alnajar S, Naushad S, Gupta RS. Genome-based phylogeny and taxonomy of the ‘Enterobacteriales’: Proposal for enterobacterales ord. nov. divided into the families Enterobacteriaceae, Erwiniaceae fam. nov., Pectobacteriaceae fam. nov., Yersiniaceae fam. nov., Hafniaceae fam. nov.,Morganellaceae fam. nov., and Budviciaceae fam. nov. Int J Syst Evol Microbiol. 2016;66:5575–99. 10.1099/ijsem.0.001485 27620848

[3] Liu H, Zhu J, Hu Q, Rao X. Morganella morganii, a non-negligent opportunistic pathogen. Int J Infect Dis. 2016;50:10–7. 10.1016/j.ijid.2016.07.006 27421818

[4] Habila Mamman P, Kazeem HM, Raji MA, Nok AJ, Kwada J, Kwaga P, et al. Int Invent J Med Med Sci. 2014.

[5] Zhao C, Tang N, Wu Y, Zhang Y, Wu Z, Li W, et al. First reported fatal Morganella morganii infections in chickens. Vet Microbiol. 2012. 10.1016/j.vetmic.2011.11.021 22176761

[6] Kilonzo-Nthenge A, Rotich E, Nahashon SN. Evaluation of drug-resistant Enterobacteriaceae in retail poultry and beef. Poult Sci. 2013;92:1098–107. 10.3382/ps.2012-02581 23472034

[7] Jones-Dias D, Clemente L, Moura IB, Sampaio DA, Albuquerque T, Vieira L, et al. Draft genomic analysis of an avian multidrug resistant morganella morganii isolate carrying qnrD1. Front Microbiol. 2016;7. 10.3389/fmicb.2016.01660 PMC5078487 27826290

[8] Chen YT, Peng HL, Shia WC, Hsu FR, Ken CF, Tsao YM, et al. Whole-genome sequencing and identification of Morganella morganii KT pathogenicity-related genes. BMC Genomics. 2012;13 Suppl 7. 10.1186/1471-2164-13-S7-S4 PMC3521468 23282187

[9] Olaitan AO, Diene SM, Gupta SK, Adler A, Assous MV, Rolain JM. Genome analysis of NDM-1 producing Morganella morganii clinical isolate. Expert Rev Anti Infect Ther. 2014;12:1297–305. 10.1586/14787210.2014.944504 25081858

[10] Minnullina L, Pudova D, Shagimardanova E, Shigapova L, Sharipova M, Mardanova A (2019). Comparative genome analysis of Uropathogenic Morganella morganii strains. Front Cell Infect Microbiol.

[11] Ishii S, Meyer KP, Sadowsky MJ. Relationship between phylogenetic groups, genotypic clusters, and virulence gene profiles of Escherichia coli strains from diverse human and animal sources. Appl Environ Microbiol. 2007;73:5703–10. 10.1128/AEM.00275-07 PMC2074926 17644637

[12] Lawson B, Franklinos LHV, Rodriguez-Ramos Fernandez J, Wend-Hansen C, Nair S, Macgregor SK, et al. Salmonella Enteritidis ST183: emerging and endemic biotypes affecting western European hedgehogs (Erinaceus europaeus) and people in Great Britain. Sci Rep. 2018;8. 10.1038/s41598-017-18667-2 PMC5799193 29402927

[13] Galac MR, Lazzaro BP. Comparative genomics of bacteria in the genus Providencia isolated from wild *Drosophila melanogaster*. BMC Genomics. 2012;13. 10.1186/1471-2164-13-612 PMC3542290 23145767

[14] Jacoby GA. AmpC Β-Lactamases. Clin Microbiol Rev. 2009;22:161–82. 10.1128/CMR.00036-08 PMC2620637 19136439

[15] Bush LM, Calmon J, Johnson CC. Newer penicillins and beta-lactamase inhibitors. Infect Dis Clin North Am. 1995;9:653–86. 7490438

[16] Carattoli A, Zankari E, Garciá-Fernández A, Larsen MV, Lund O, Villa L, et al. In Silico detection and typing of plasmids using plasmidfinder and plasmid multilocus sequence typing. Antimicrob Agents Chemother. 2014;58:3895–903. 10.1128/AAC.02412-14 PMC4068535 24777092

[17] Parks DH, Imelfort M, Skennerton CT, Hugenholtz P, Tyson GW. CheckM: Assessing the quality of microbial genomes recovered from isolates, single cells, and metagenomes. Genome Res. 2015;25:1043–55. 10.1101/gr.186072.114 PMC4484387 25977477

[18] Jensen KT, Frederiksen W, Hickman-Brenner FW, Steigerwalt AG, Riddle CF, Brenner DJ. Recognition of Morganella Subspecies, with Proposal of Morganella morganii subsp. morganii subsp. nov. and Morganella morganii subsp. sibonii subsp. nov. Int J Syst Bacteriol. 1992;42:613–20. 10.1099/00207713-42-4-613 1390112

[19] Hedge J, Wilson DJ. Bacterial phylogenetic reconstruction from whole genomes is robust to recombination but demographic inference is not. MBio. 2014;5. 10.1128/mBio.02158-14 PMC4251999 25425237

[20] Minnullina L, Pudova D, Shagimardanova E, Shigapova L, Sharipova M, Mardanova A. Comparative genome analysis of uropathogenic morganella morganii strains. Front Cell Infect Microbiol. 2019. 10.3389/fcimb.2019.00167. 10.3389/fcimb.2019.00167 PMC6558430 31231616

[21] Jones BD, Mobley HLT. Genetic and biochemical diversity of ureases of Proteus, Providencia, and Morganella species isolated from urinary tract infection. Infect Immun. 1987;55:2198–203. 10.1128/iai.55.9.2198-2203.1987 PMC260678 3623698

[22] Willis LM, Whitfield C. Structure, biosynthesis, and function of bacterial capsular polysaccharides synthesized by ABC transporter-dependent pathways. Carbohydr Res. 2013;378:35–44. 10.1016/j.carres.2013.05.007 23746650

[23] Rancurel C, Legrand L, Danchin EGJ. Alienness: rapid detection of candidate horizontal gene transfers across the tree of life. Genes (Basel). 2017. 10.3390/genes8100248 PMC5664098 28961181

[24] Cosentino S, Voldby Larsen M, Møller Aarestrup F, Lund O. PathogenFinder - Distinguishing Friend from Foe Using Bacterial Whole Genome Sequence Data. PLoS One. 2013;8. 10.1371/journal.pone.0077302 PMC3810466 24204795

[25] Casjens S. Prophages and bacterial genomics: what have we learned so far? Mol Microbiol. 2003;49:277–300. 10.1046/j.1365-2958.2003.03580.x 12886937

[26] Gillings MR. Integrons: past, present, and future. Microbiol Mol Biol Rev. 2014;78:257–77. 10.1128/MMBR.00056-13 PMC4054258 24847022

[27] Guo X, Rao Y, Guo L, Xu H, Lv T, Yu X, et al. Detection and genomic characterization of a morganella morganiiisolate from China that produces NDM-5. Front Microbiol. 2019. 10.3389/fmicb.2019.01156 PMC6546717 31191484

[28] Mbelle N, Osei Sekyere J, Feldman C, Maningi NE, Modipane L, Essack SY. Genomic analysis of two drug-resistant clinical Morganella morganii strains isolated from UTI patients in Pretoria, South Africa. Lett Appl Microbiol. 2020;70:21–8. 10.1111/lam.13237 31630429

[29] Mahrouki S, Bourouis A, Chihi H, Ouertani R, Ferjani MB, Moussa M, et al. First characterisation of plasmid-mediated quinolone resistance-qnrS1 co-expressed Bla CTX-M-15 and Bla DHA-1 genes in clinical strain of Morganella morganii recovered from a Tunisian intensive care unit. Indian J Med Microbiol. 2012;30:437–41. 10.4103/0255-0857.103765 23183469

[30] Hacker J, Kaper JB. Pathogenicity Islands and the Evolution of Microbes. Annu Rev Microbiol. 2000;54:641–79. 10.1146/annurev.micro.54.1.641 11018140

[31] Kanehisa M, Sato Y, Morishima K. BlastKOALA and GhostKOALA: KEGG Tools for Functional Characterization of Genome and Metagenome Sequences. J Mol Biol. 2016;428:726–31. 10.1016/j.jmb.2015.11.006 26585406

[32] Wood JM, Bremer E, Csonka LN, Kraemer R, Poolman B, Van der Heide T (2001). Osmosensing and osmoregulatory compatible solute accumulation by bacteria. Comparative Biochemistry and Physiology - A Molecular and Integrative Physiology.

[33] Wargo MJ. Homeostasis and catabolism of choline and glycine betaine: lessons from Pseudomonas aeruginosa. Appl Environ Microbiol. 2013;79:2112–20. 10.1128/AEM.03565-12 PMC3623244 23354714

[34] Rahman A, Bhuiyan OF, Sadique A, Afroze T, Sarker M, Momen AMI, et al. Whole genome sequencing provides genomic insights into three Morganella morganii strains isolated from bovine rectal swabs in Dhaka, Bangladesh. FEMS Microbiol Lett. 2019;367. 10.1093/femsle/fnaa043 32129839

[35] Bradford PA. Extended-spectrum β-lactamases in the 21st century: characterization, epidemiology, and detection of this important resistance threat. Clin Microbiol Rev. 2001;14:933–51. 10.1128/CMR.14.4.933-951.2001 PMC89009 11585791

[36] Servin AL. Pathogenesis of Afa/Dr diffusely adhering Escherichia coli. Clin Microbiol Rev. 2005;18:264–92. 10.1128/CMR.18.2.264-292.2005 PMC1082796 15831825

[37] Wurpel DJ, Beatson SA, Totsika M, Petty NK, Schembri MA (2013). Chaperone-usher fimbriae of Escherichia coli. PLoS One.

[38] Spurbeck RR, Dinh PC, Walk ST, Stapleton AE, Hooton TM, Nolan LK (2012). *Escherichia coli* Isolates That Carry vat, fyuA, chuA, and yfcV Efficiently Colonize the Urinary Tract. Infect Immun.

[39] Easton DM, Allsopp LP, Phan MD, Moriel DG, Goh GK, Beatson SA, et al. The intimin-like protein FdeC is regulated by H-NS and temperature in enterohemorrhagic *Escherichia coli*. Appl Environ Microbiol. 2014;80:7337–47. 10.1128/AEM.02114-14 PMC4249167 25239893

[40] Bai X, Fu S, Zhang J, Fan R, Xu Y, Sun H (2018). Identification and pathogenomic analysis of an Escherichia coli strain producing a novel Shiga toxin 2 subtype. Sci Rep.

[41] Andrade A, Pardo JP, Espinosa N, Perez-Hernandez G, Gonzalez-Pedrajo B (2007). Enzymatic characterization of the enteropathogenic Escherichia coli type III secretion ATPase EscN. Arch Biochem Biophys.

[42] Chatterjee A, Caballero-Franco C, Bakker D, Totten S, Jardim A (2015). Pore-forming activity of the Escherichia coli type III secretion system protein EspD. J Biol Chem.

[43] Morgan JAW, Sergeant M, Ellis D, Ousley M, Jarrett P. Sequence analysis of insecticidal genes from Xenorhabdus nematophilus PMFI296. Appl Environ Microbiol. 2001;67:2062–9. 10.1128/AEM.67.5.2062-2069.2001 PMC92837 11319082

[44] Alispahic M, Christensen H, Bisgaard M, Hess M, Hess C. MALDI-TOF mass spectrometry confirms difficulties in separating species of the Avibacterium genus. Avian Pathol. 2014;43:258–63. 10.1080/03079457.2014.916038 24802229

[45] Bauer AW, Kirby WM, Sherris JC, Turck M. Antibiotic susceptibility testing by a standardized single disk method. Am J Clin Pathol. 1966;493–6. 5325707

[46] Seemann T. Prokka: rapid prokaryotic genome annotation. Bioinformatics. 2014;30:2068–9. 10.1093/bioinformatics/btu153 24642063

[47] Orlek A, Phan H, Sheppard AE, Doumith M, Ellington M, Peto T, et al. A curated dataset of complete Enterobacteriaceae plasmids compiled from the NCBI nucleotide database. Data Br. 2017. 10.1016/j.dib.2017.04.024 PMC5426034 28516137

[48] Song W, Sun HX, Zhang C, Cheng L, Peng Y, Deng Z, et al. Prophage hunter: an integrative hunting tool for active prophages. Nucleic Acids Res. 2019;47:W74–80. 10.1093/nar/gkz380 PMC6602508 31114893

[49] Cury J, Jové T, Touchon M, Néron B, Rocha EP. Identification and analysis of integrons and cassette arrays in bacterial genomes. Nucleic Acids Res. 2016;44:4539–50. 10.1093/nar/gkw319 PMC4889954 27130947

[50] Bertelli C, Laird MR, Williams KP, Lau BY, Hoad G, Winsor GL, et al. IslandViewer 4: expanded prediction of genomic islands for larger-scale datasets. Nucleic Acids Res. 2017;45:W30–5. 10.1093/nar/gkx343 PMC5570257 28472413

[51] Treangen T, Ondov B, Koren S, Phillippy A. Rapid Core-Genome Alignment and Visualization for Thousands of Intraspecific Microbial Genomes. bioRxiv. 2014;007351. 10.1186/s13059-014-0524-x PMC4262987 25410596

[52] Lakin SM, Dean C, Noyes NR, Dettenwanger A, Ross AS, Doster E, et al. MEGARes: An antimicrobial resistance database for high throughput sequencing. Nucleic Acids Res. 2017;45:D574–80. 10.1093/nar/gkw1009 PMC5210519 27899569

[53] Steinegger M, Söding J. MMseqs2 enables sensitive protein sequence searching for the analysis of massive data sets. Nat Biotechnol. 2017;35:1026–8. 10.1038/nbt.3988 29035372

[54] Löytynoja A. Phylogeny-aware alignment with PRANK. Methods Mol Biol. 2014;1079:155–70. 10.1007/978-1-62703-646-7_10 24170401

[55] Yang Z. PAML 4: phylogenetic analysis by maximum likelihood. Mol Biol Evol. 2007;24:1586–91. 10.1093/molbev/msm088 17483113

[56] Kanehisa M (2003). The KEGG Database.

