# Supplementary material for: Sequencing of five poultry strains elucidates phylogenetic relationships and divergence in virulence genes in Morganella morganii
Source: BMC Genomics. 2020 Aug 24;21:579. doi: 10.1186/s12864-020-07001-2 (PMC7446228; doi:10.1186/s12864-020-07001-2)
Supplement: Supplementary file 1 — Additional file 1: Table S1. MICRONAUT antimicrobial substances and concentrations. [file 12864_2020_7001_MOESM1_ESM.docx]

| **Class** | **Antimicrobial substance** | **Concentrations (µg/ml)** | | | | | | | | | |
| --- | --- | --- | --- | --- | --- | --- | --- | --- | --- | --- | --- |
| Cephalosporins 4^th^ generation | Cefepime | 128 | 64 | 32 | 16 | 8 | 4 | 2 | 1 |  |  |
|  | Cefepime / clavulanic acid | 32/4 | 16/4 | 8/4 | 4/4 | 2/4 | 1/4 | 0.5/4 | 0.25/4 |  |  |
| Cephalosporins 3^th^ generation | Ceftazidime | 128 | 64 | 32 | 16 | 8 | 4 | 2 | 1 |  |  |
|  | Ceftazidime / clavulanic acid | 32/4 | 16/4 | 8/4 | 4/4 | 2/4 | 1/4 | 0.5/4 | 0.25/4 |  |  |
|  | Cefotaxime | 128 | 64 | 32 | 16 | 8 | 4 | 2 | 1 |  |  |
|  | Cefotaxime / clavulanic acid | 32/4 | 16/4 | 8/4 | 4/4 | 2/4 | 1/4 | 0.5/4 | 0.25/4 |  |  |
|  | Ceftazidime / 3-APB | 32 | 16 | 8 | 4 | 2 | 1 | 0.5 | 0.25 |  |  |
|  | Cefotaxime / 3-APB | 32 | 16 | 8 | 4 | 2 | 1 | 0.5 | 0.25 |  |  |
| Carbapenems | Meropenem | 128 | 64 | 32 | 16 | 8 | 4 | 2 | 1 | 0.25 | 0.125 |
|  | Meropenem / 3-APB | 32 | 16 | 8 | 4 | 2 | 1 | 0.5 | 0.25 |  |  |
|  | Meropenem / EDTA | 32 | 16 | 8 | 4 | 2 | 1 | 0.5 | 0.25 |  |  |
|  | Ertapenem | 0.5 | 0.25 | 0.125 |  |  |  |  |  |  |  |
| Penicillins | Temocillin | 128 | 32 |  |  |  |  |  |  |  |  |

**Supplementary Table 1 -** MICRONAUT antimicrobial substances and concentrations
