# Supplementary figures and images for: Sequencing of five poultry strains elucidates phylogenetic relationships and divergence in virulence genes in Morganella morganii
Source: BMC Genomics. 2020 Aug 24;21:579. doi: 10.1186/s12864-020-07001-2 (PMC7446228; doi:10.1186/s12864-020-07001-2)

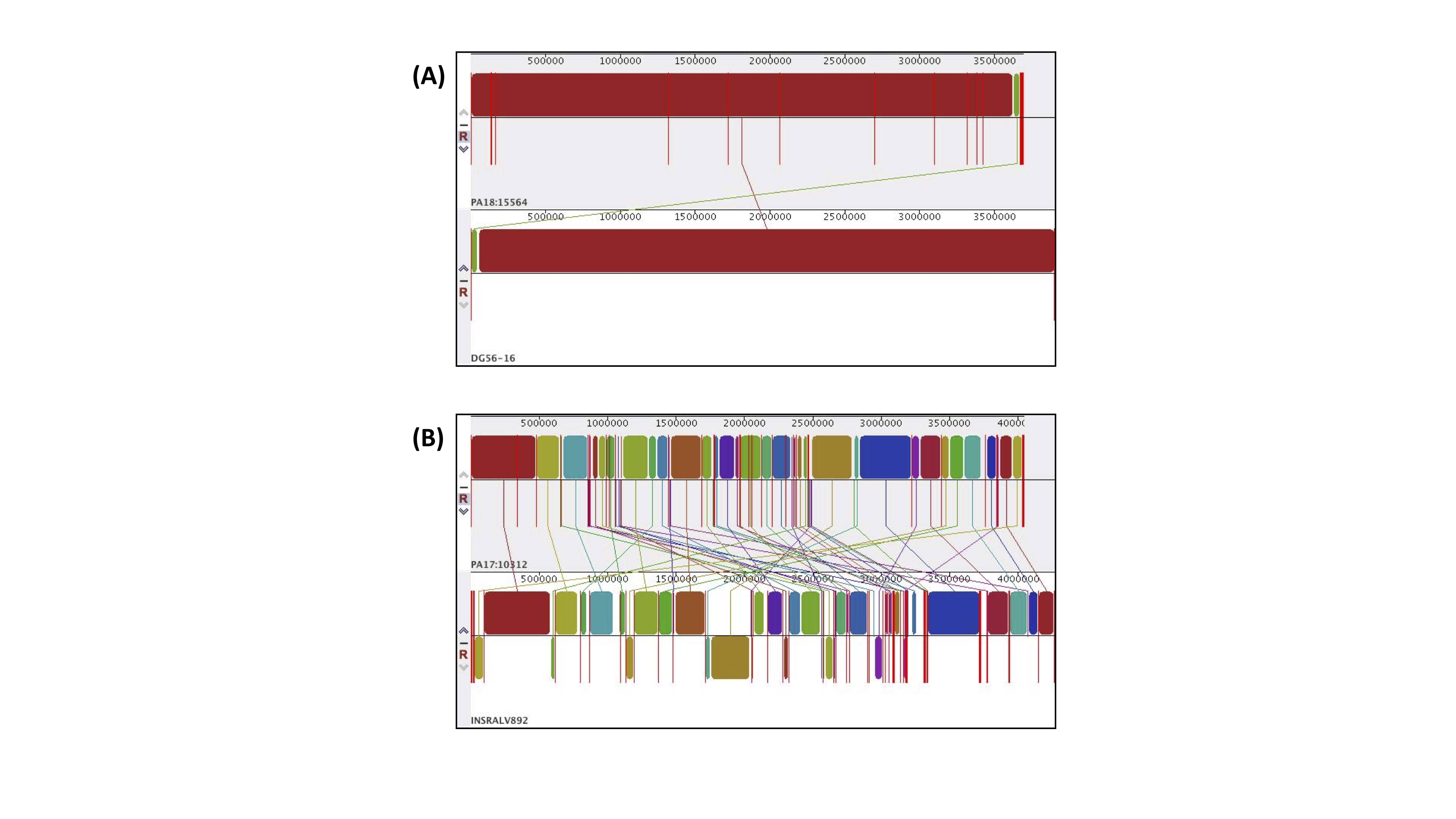

Supplement: Supplementary file 2 — Additional file 2: Figure S1. Genomic alignment computed with MAUVE between two of the sequenced poultry strains and the closest strain on the tree shown in Fig. 2: (A) PA18/15564 aligned to the DG56–16 strain, (B) PA17/10312 aligned to the INSRALV892 strain. [file 12864_2020_7001_MOESM2_ESM.jpeg]

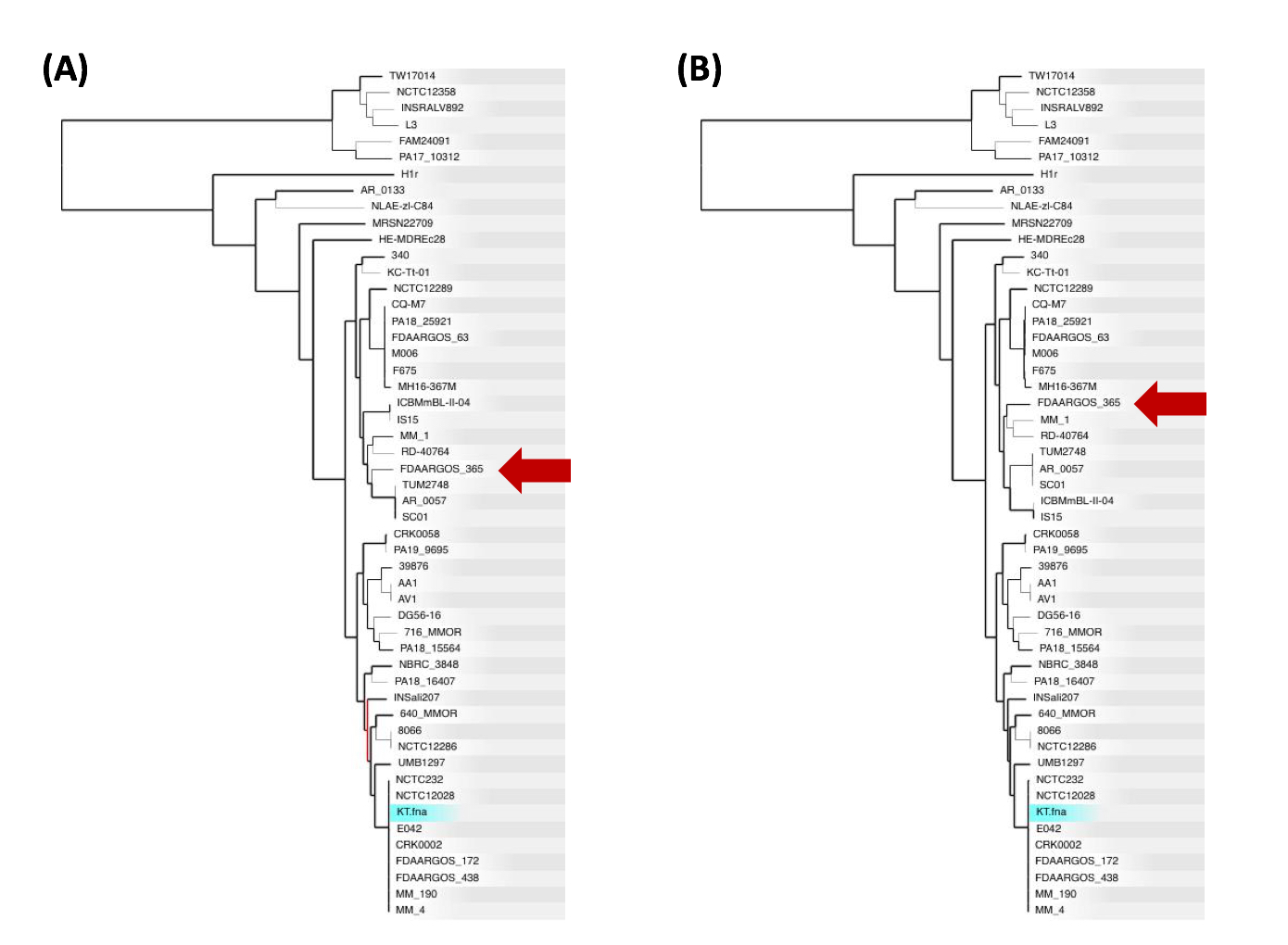

Supplement: Supplementary file 3 — Additional file 3: Figure S2. A) Tree of the 52 M. morganii strains analyzed in this study derived by alignment of the core genome using the program parsnp. The KT strain was used as a reference for the multiple genome alignment. B) The same tree but corrected for recombination. The red arrows indicate the cluster that shows differences between the two trees. [file 12864_2020_7001_MOESM3_ESM.jpeg]

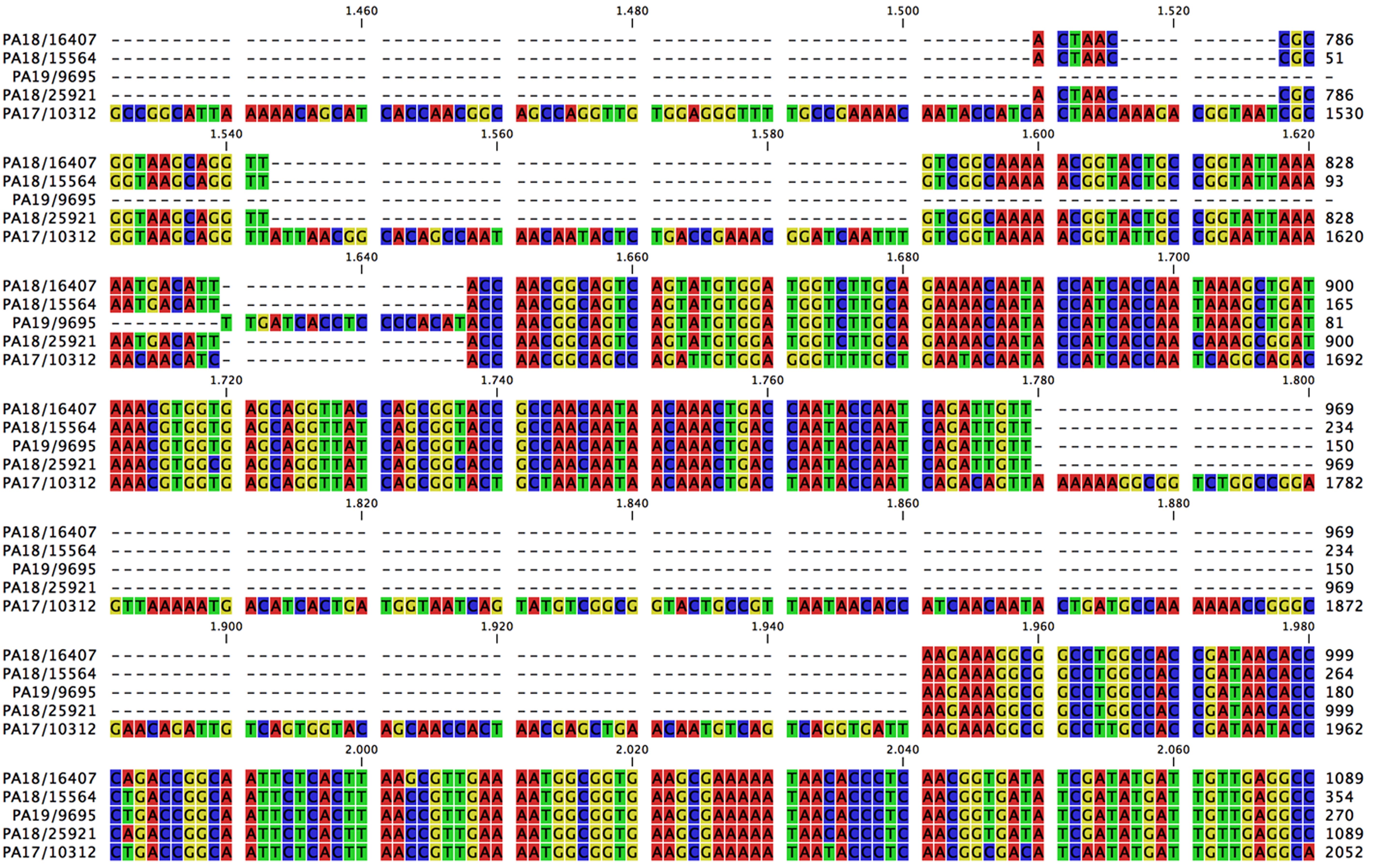

Supplement: Supplementary file 4 — Additional file 4: Figure S3. Multiple alignment of an internal region of the RtxA toxin in the five poultry strains showing high sequence divergence. [file 12864_2020_7001_MOESM4_ESM.jpeg]

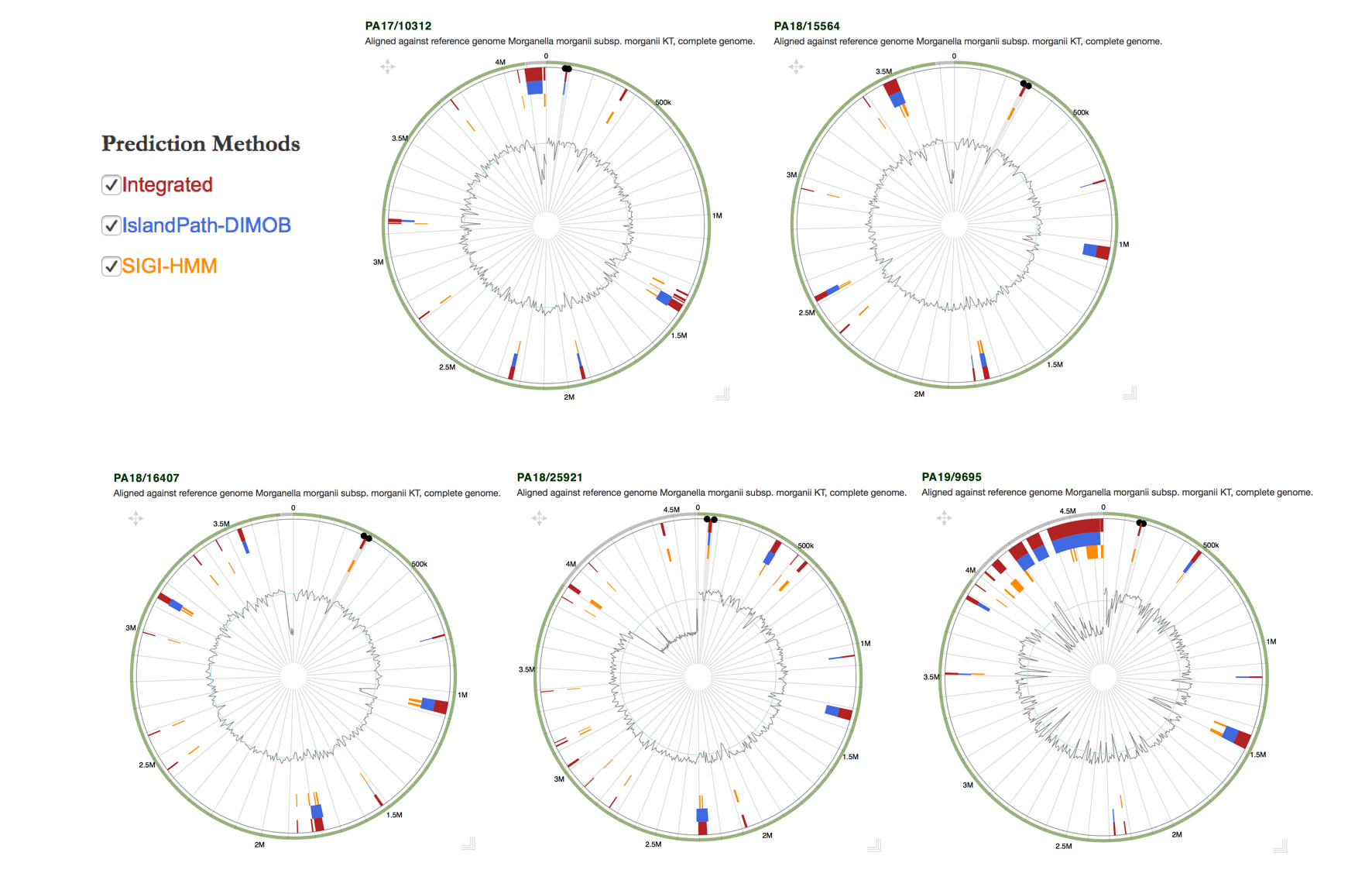

Supplement: Supplementary file 8 — Additional file 8: Figure S4. Genomic visualization of pathogenicity islands in the five sequenced poultry strains, performed in this study. [file 12864_2020_7001_MOESM8_ESM.jpeg]

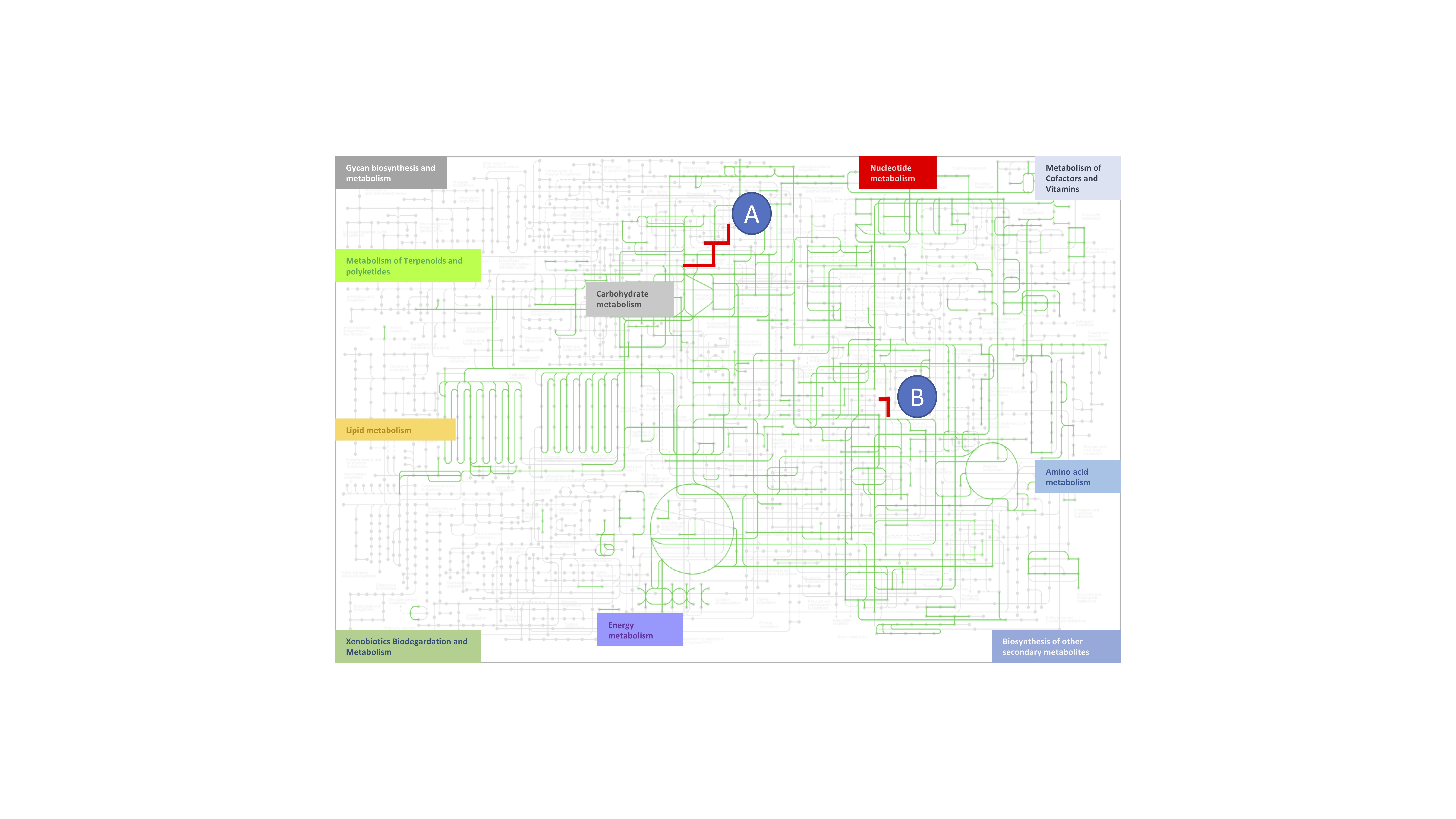

Supplement: Supplementary file 9 — Additional file 9: Figure S5. Metabolic network of M. morganii strain PA17/10312 (computed through the web interface of BlastKOALA [31] in this study) showing two novel pathways specific to this strain: (A) Sucrose catabolism, sucrose = > glucose, (B) Betaine biosynthesis, betaine = > choline. [file 12864_2020_7001_MOESM9_ESM.jpeg]
