# Supplementary material for: Sequencing of five poultry strains elucidates phylogenetic relationships and divergence in virulence genes in Morganella morganii
Source: BMC Genomics. 2020 Aug 24;21:579. doi: 10.1186/s12864-020-07001-2 (PMC7446228; doi:10.1186/s12864-020-07001-2)
Supplement: Supplementary file 5 — Additional file 5: Table S2. List of candidate genes in the PA17/10312 strain with evidence for horizontal gene transfer from closely-related Morganellaceae species. [file 12864_2020_7001_MOESM5_ESM.docx]

**Supplementary Table 2** – List of candidate genes in the PA17/10312 strain with evidence for horizontal gene transfer from closely-related *Morganellaceae* species

| **GeneID PA17/10312** | **Gene description** | **Alien Index (AI)** | **Putative donor species** |
| --- | --- | --- | --- |
| IECFJLGK_02819 | Invasion protein InvA | 460,52 | Arsenophonus nasoniae |
| IECFJLGK_00013 | hypothetical protein | 436,21 | Morganella psychrotolerans |
| IECFJLGK_00431 | hypothetical protein | 294,28 | Morganella psychrotolerans |
| IECFJLGK_02809 | Yop proteins translocation protein U | 196,02 | Morganella psychrotolerans |
| IECFJLGK_00255 | putative fimbrial-like protein | 122,26 | Proteus genome sp. |
| IECFJLGK_01234 | putative fimbrial chaperone YraI | 62,03 | Providencia heimbachae |
| IECFJLGK_03583 | Shiga toxin 2 subunit A | 48,47 | Morganella psychrotolerans |
