# Supplementary material for: Sequencing of five poultry strains elucidates phylogenetic relationships and divergence in virulence genes in Morganella morganii
Source: BMC Genomics. 2020 Aug 24;21:579. doi: 10.1186/s12864-020-07001-2 (PMC7446228; doi:10.1186/s12864-020-07001-2)
Supplement: Supplementary file 6 — Additional file 6: Table S3. Annotation of active prophages in the five sequenced poultry strains. [file 12864_2020_7001_MOESM6_ESM.docx]

**Supplementary Table 3** − Annotation of prophages in the five sequenced poultry strains

| **Strain** | **Contig** | **Contig Start** | **Contig End** | **Prophage** |
| --- | --- | --- | --- | --- |
| PA17/10312 | contig_1 | 725087 | 753336 | Morganella phage IME1369_01 |
| PA17/10312 | contig_19 | 343 | 13233 | N/A |
| PA17/10312 | contig_19 | 4065 | 15839 | N/A |
| PA17/10312 | contig_5 | 105339 | 122288 | N/A |
| PA18/15564 | contig_2 | 1491 | 22710 | Morganella phage IME1369_02 |
| PA18/15564 | contig_7 | 244 | 22187 | Morganella phage IME1369_01 |
| PA18/15564 | contig_7 | 10129 | 20145 | Burkholderia phage BcepB1A |
| PA18/16407 | contig_1 | 604611 | 648422 | Morganella phage IME1369_01 |
| PA18/16407 | contig_1 | 630307 | 649246 | Morganella phage IME1369_01 |
| PA18/25921 | contig_1 | 716 | 42414 | Morganella phage IME1369_01 |
| PA18/25921 | contig_16 | 154290 | 168892 | Salmonella phage FO1a |
| PA18/25921 | contig_16 | 153584 | 168102 | Salmonella phage FO1a |
| PA18/25921 | contig_2 | 390968 | 432562 | Morganella phage IME1369_01 |
| PA18/25921 | contig_2 | 471777 | 520136 | Morganella phage IME1369_01 |
| PA19/9695 | contig_45 | 1115 | 32650 | Morganella phage IME1369_02 |
