# Supplementary material for: Sequencing of five poultry strains elucidates phylogenetic relationships and divergence in virulence genes in Morganella morganii
Source: BMC Genomics. 2020 Aug 24;21:579. doi: 10.1186/s12864-020-07001-2 (PMC7446228; doi:10.1186/s12864-020-07001-2)
Supplement: Supplementary file 10 — Additional file 10: Table S5. List of M. morganii strains used for the phylogenetic analysis with their respective NCBI accession IDs. [file 12864_2020_7001_MOESM10_ESM.docx]

**Supplementary Table 5** − List of *M. morganii* strains used for the phylogenetic analysis with their respective NCBI accession IDs

| **Strain** | **GenBank ID** |  | **Strain** | **GenBank ID** |
| --- | --- | --- | --- | --- |
| 8066 | GCA_000966695.1 |  | MH16-367M | GCA_003114875.2 |
| NCTC12286 | GCA_900453165.1 |  | CQ-M7* | GCA_003931985.1 |
| KT | GCA_000286435.2 |  | NCTC12289 | GCA_900453195.1 |
| NCTC12028 | GCA_900478755.1 |  | MM_1 | GCA_003390295.1 |
| NCTC232 | GCA_900453135.1 |  | RD-40764 | GCA_003852695.1 |
| MM_4 | GCA_003340585.1 |  | AR_0057 | GCA_002968775.1 |
| FDAARGOS_172 | GCA_001558895.2 |  | SC01 | GCA_000307755.2 |
| MM_190 | GCA_003287815.1 |  | TUM2748 | GCA_003176395.1 |
| FDAARGOS_438 | GCA_002588265.1 |  | ICBMmBL-II-04 | GCA_002029935.1 |
| E042 | GCA_002416605.1 |  | IS15 | GCA_000530115.1 |
| CRK0002 | GCA_002184465.2 |  | FDAARGOS_365 | GCA_002386305.1 |
| 640_MMOR | GCA_001066745.1 |  | KC-Tt-01 | GCA_002891475.1 |
| UMB1297 | GCA_002847885.1 |  | 340 | GCA_000747035.1 |
| INSali207 | GCA_001653675.1 |  | HE-MDREc28 | GCA_003730795.1 |
| NBRC_3848 | GCA_001598895.1 |  | MRSN22709 | GCA_000770295.1 |
| AV1 | GCA_002077705.1 |  | NLAE-zl-C84 | GCA_900142745.1 |
| AA1 | GCA_002077675.1 |  | AR_0133 | GCA_003071325.1 |
| 39876 | GCA_002180575.1 |  | H1r | GCA_000633515.1 |
| DG56-16 | GCA_003573445.1 |  | INSRALV892 | GCA_001263435.1 |
| 716_MMOR | GCA_001066005.1 |  | L3 | GCA_001006565.1 |
| CRK0058 | GCA_002185325.2 |  | TW17014 | GCA_001274995.1 |
| FDAARGOS_63 | GCA_000783955.2 |  | NCTC12358 | GCA_900453145.1 |
| M006 | GCA_002417235.1 |  | FAM24091 | GCA_003034205.1 |
| F675 | GCA_000752335.1 |  |  |  |

*Excluded from RefSeq: contaminated, genome length too large
